# The time of Calcium Feeding Affects the Productive Performance of Sows

**DOI:** 10.3390/ani9060337

**Published:** 2019-06-10

**Authors:** Lumin Gao, Xue Lin, Chunyan Xie, Tianyong Zhang, Xin Wu, Yulong Yin

**Affiliations:** 1 Key Laboratory of Agro-ecological Processes in Subtropical Region, Institute of Subtropical Agriculture, Chinese Academy of Sciences; National Engineering Laboratory for Pollution Control and Waste Utilization in Livestock and Poultry Production; Hunan Provincial Engineering Research Center for Healthy Livestock and Poultry Production, Changsha 410125, China; gao-lumin@foxmail.com (L.G.); risca@tanke.com.cn (X.L.); yinyulong@isa.ac.cn (Y.Y.); 2 Hunan Co-Innovation Center of Safety Animal Production, College of Animal Science and Technology; College of Bioscience and Biotechnology, Hunan Agricultural University, Changsha 410128, China; xiechunyan@hunau.edu.cn; 3 Henan Guang’an Biology Technology Co. Ltd., Zhengzhou 450001, China; ztyd2009@163.com

**Keywords:** calcium, sow, mineral element profile, umbilical serum, milk

## Abstract

**Simple Summary:**

Calcium (Ca) is an important factor that affects the reproductive and lactation performance of sows. Circadian clocks and nutrient metabolism interact. We found that maternal feeding of Ca, varying with feeding time, improved the profile of some mineral elements in umbilical serum and milk, which contribute toward improving the productive performance of sows. This study confirmed that the absorption and metabolism of Ca in mammals also have circadian rhythms.

**Abstract:**

This study aims to investigate the effect of Calcium (Ca) feeding time on a sow’s productive performance and the profiles of serum mineral elements during late pregnancy and lactation. A total of 75 pregnant sows were assigned to three groups: The control (C), earlier-later (E-L), and later-earlier (L-E) groups. During late pregnancy, the C group was fed an extra 4.5 g Ca (in the form of CaCO_3_) at both 06:00 and 15:00, the E-L group was fed an extra 9 g Ca at 06:00, and the L-E group was fed an extra 9 g Ca at 15:00. Similar treatments with double the amount of Ca were applied during lactation. The results show that, compared with the C group, L-E feeding decreased the number of stillbirths and the duration of farrowing and placenta expulsion (FARPLA) and increased the average daily weight gain (ADG) of piglets. Similarly, E-L feeding increased the ADG of piglets (*p* < 0.05). Furthermore, both E-L and L-E feeding increased the Ca levels in sow serum and umbilical serum, and the Fe levels in umbilical serum, but decreased the Ca levels in the placenta and colostrum (*p* < 0.05). Experiments on the genes involved in mineral element transport showed that E-L feeding activated the mRNA expression of TRPV5, S100G, SLC30A7, SLC39A4, and Ferroportin1, while it inhibited the mRNA expression of ATP7A in the placenta (*p* < 0.05). Moreover, L-E feeding up-regulated the mRNA expression of ATP2B and IREB2, while it down-regulated the mRNA expression of ATP7B in the placenta (*p* < 0.05). In conclusion, the present study demonstrated that maternal Ca feeding at 15:00 h during late pregnancy and lactation decreased FARPLA and stillbirths and improved the growth performance of suckling piglets by altering the mineral element of the metabolism in the umbilical serum and milk, compared to conventional feeding regimes.

## 1. Introduction

Calcium (Ca) demands are understandably greater in modern sow lines with larger litter sizes and greater milk production capabilities. It has been reported that dietary Ca plays an important role in the skeletal development of piglets during late pregnancy [1]. Notably, the Ca level in the diet also affects milk Ca and other mineral element concentrations [2]. Thus, higher mineral requirements, particularly for Ca, during later pregnancy may be a limiting factor in colostrum synthesis in mammary glands [3]. High prolificacy and prolonged farrowing in sows have been linked to hypocalcemic responses during farrowing; therefore, low levels or a low utilization rate of Ca in the diet fed to gestating sows can reduce litter size, prolong delivery time, increase the number of stillbirths, and result in a higher occurrence of skeletal problems in piglets [4]. Consequently, improving the utilization of Ca and other mineral elements is important for the productive performance of sows.

The feeding of nutrients to animals must be coordinated with endogenous physiological rhythms to optimize mammalian production [5], signifying the importance of feeding time. On the one hand, recent studies show that circadian clocks and energy metabolism interact, and nutrient utilization is affected by the time of feed intake even when the same type of feed and the same number of calories are consumed [6,7]. One study shows that feeding with a high energy diet in the evening increases the risk of obesity more than feeding with a high energy diet before 12:00, and that evening or night feeding increases milk fat synthesis and energy production [8]. In addition, feeding pregnant sows at 15:30 will alter their energy and nutrient metabolism and improve their backfat (BF) thickness gain compared with conventional feeding regimes [9], and crude protein feeding time modulates the lipid profiles in sow milk and plasma [10]. On the other hand, the mRNA expression of circadian clocks in livestock is also affected by feeding time [11], and one study also reported that Ca feeding time in laying hens affected the expression of genes related to the circadian clock and Ca transporters and led to circadian variations in serum Ca [12]. Ca feeding time during late pregnancy affects fatty acid transport and circadian clock-related changes in the placenta of pigs [13]. Overall, it was speculated that daily Ca feeding time may affect productive performance and mineral element metabolism in sows. However, there are few reports on such effects. Therefore, this study focuses on the productive performance of sows, the mineral element profiles of their milk and serum, and their metal transporters under conditions of daily dietary Ca feeding at 06:00 and 15:00.

## 2. Material and Methods

### 2.1. Ethics Statement

The animal experiments were approved by the Animal Care and the Animal Welfare Committees of the Institute of Subtropical Agriculture at the Chinese Academy of Sciences (2015-8A).

### 2.2. Animals and Housing

A total of 75 pregnant sows (Large White × Landrace) on d 85 ± 3 of pregnancy with similar parity (3–6 parities) and close parturition dates (back-fat thickness 15–17 mm) were randomly divided into three dietary treatments (the control (C) group, the earlier-later (E-L) group, and the later-earlier (L-E) group) with 25 replicates in each.

Sows were housed in farrowing accommodation from day 85 ± 3 of gestation to the date of weaning with full-leakage slats, limited movement, and free access to drinking water. Disease prevention, health care, and management were consistent with the farm. Experiments were carried out between June and August by Henan Guang’an Biology Technology Co. Ltd. (Zhengzhou, 450001, China).

### 2.3. Animal Treatment and Feeding

A single factor randomized design was adopted for the experiment. Housing and breeding management were performed as described recently in detail [14]. The experiment started on day 85 of gestation and ended on day 14 of lactation. Sows were fed corn-soybean-based diets, and all nutrients met the 2012 National Research Council nutrient recommendations for sows. The ingredient composition and nutrient content of the basic diets (0.30% Ca) are shown in Table 1. 

According to Xin Wu, the appropriate amount of Ca in a late pregnancy and lactation diet for sows is 0.75% [15]. As the average feed intake of sows in late pregnancy is 2 kg/d and the average feed intake in lactating sows is 4 kg/d on this experiment farm, extra Ca is required in the daily diet of every sow. The experimental period was divided into two stages: Late pregnancy and lactation. During late pregnancy, the C group was fed an extra 4.5 g Ca (in the form of CaCO_3_) at both 06:00 and 15:00, the E-L group was fed an extra 9 g Ca at 06:00, and the L-E group was fed an extra 9 g Ca at 15:00. During lactation, the C group was fed an extra 9 g Ca at both 06:00 and 15:00, the E-L group was fed an extra 18 g Ca at 06:00, and the L-E group was fed an extra 18 g Ca at 15:00. Sows were fed equal quantities of feed at 06:00 and 15:00 throughout the experiment to ensure that they all consumed equal amounts of feed with the same daily Ca content. Daily intake was recorded and all feed was consumed. Every sow was fed 2.5–3.0 kg/d from day 85 to day 107 of pregnancy, 1.8 kg/d from 5 days before delivery, 1.0–1.5 kg/d on the day of delivery, and 3–5 kg/d after delivery. 

A total of 20 sows with litter sizes between 10 and 13 were selected from each group for the lactation experiment. Litters were standardized to 11 piglets within 24 h after farrowing by removing piglets from sows with >11 piglets and placing them with sows of the same group with <11 piglets [16]. The other fifteen litters were kept as reserves (five litters for each group). If a piglet in an experimental group died within 7 days, a piglet from a reserve litter would be added, but no more than two piglets were added per litter. Piglets were weighed within 6 h of birth, on day 1 and day 14 of lactation. The number of healthy and dead piglets was recorded during the whole lactation period to obtain the survival rate.

### 2.4. Sample Collection

#### 2.4.1. The Feed Intake and Back-Fat Thickness of Sows

The feed intake of sows was recorded daily during the experiment. In addition, back-fat thickness was measured at P2 (6 cm from the mid line at the head of the last rib) on both the left and right sides with an ultrasonic device (Agroscan A16, France) on day 85 after pregnancy, and days 1 and 14 of lactation. The average of BF was calculated and recorded.

#### 2.4.2. The Productive Performance of Sows

Farrowing was not induced, and no attempts were made to interfere with the natural delivery of the piglets. The reproductive performance of the sows, such as the litter size and number of stillbirths, was recorded at delivery. The exact moments of birth and the duration of farrowing and placenta expulsion (FARPLA, defined as the time interval between the birth of the first piglet and the expulsion of the last placenta) were registered [17]. The birth weight of the piglets was recorded for identification within 6 h after birth. Intra-uterine growth restriction (IUGR) refers to the impaired growth and development of a mammalian embryo or fetus or its organs during pregnancy [18]. Neonatal piglets with birth weights near the mean birth weight (±0.5 standard deviation (SD)) were identified as having normal birth weights, whereas piglets with at least 1.5 SD lower birth weight were defined as suffering from IUGR [19]. In addition, the litter size was standardized to 10 pigs per sow through cross-fostering within 24 h, piglets were weighed on the 1st, 7th, and 14th day of life, and daily gains were calculated [16].

### 2.5. Serum Samples

Blood samples (5 mL) were taken from nine sows per group from the jugular vein and umbilical cord on farrowing day, and from the jugular vein on the 7th day of lactation, both at 12:00 and 24:00. The blood samples were centrifuged at 3000× *g* for 10 min at 4 °C to obtain serum samples and then stored at −20 °C for subsequent trace mineral analysis. 

### 2.6. Milk Samples

Colostrum and milk samples (50 mL) were collected from 4–6 mammary glands of nine sows, marked above per group by hand milking during the delivery, and on the 7th day of lactation, and milk samples were taken on the 7th day of lactation 2 times a day (12:00 and 24:00) by hand milking, and 10 to 30 uL oxytocin (Oxytocin Injection, Jiangxi Huiqifeng Biology Technology Co. Ltd., China) was injected before milking. The milk was aliquoted and frozen at −70 °C for 5 min and then stored at −20 °C until it was assayed [16].

### 2.7. Placenta Samples

Immediately after farrowing, two allantochorion tissue samples were obtained from similar areas (large vessels were avoided) of the placentas of piglets with birth weights of about 1.5 kg from the 27 sows marked above (*n* = 9). About 10 g of placenta tissue was stored in liquid nitrogen for quantitative real time-polymerase chain reaction (RT-PCR) analyses. The rest of the placenta tissue was stored at −20 °C for mineral element analyses [16].

### 2.8. Mineral Element Determination in Serum, Milk, and Placenta Samples

The concentrations of Ca, Fe, Cu, and Zn were determined in the placenta, serum, and milk of sows with an inductively coupled plasma (ICP) emission spectrometer [20]. Placenta samples were completely dried at 60 °C and ground into powder before pretreatment. Thereafter, the placenta (1.00 ± 0.05 g), milk, and serum (each 1.00 ± 0.05 mL) samples were weighed in triplicate and subjected to acid-digestion using a mixture of nitric and perchloric acids under the following heating procedure: 80 °C, 60 min; 120 °C, 30 min; and 180 °C, 30 min. The samples were dried at 260 °C and re-dissolved with 5 mL of 1% HNO_3_. All samples were then transferred to 25 mL volumetric flasks and diluted several times with 1% HNO_3_ based on the concentration of the sample. Finally, they were filtered with a suitable standard solution and submitted for ICP analyses (ICP-720ES, Agilent, Palo Alto, CA, USA). In addition, two blank absorbance values were used throughout the analysis. All results were certified using standard references and the precision of the analytical method, calculated as the relative standard deviation of the metal concentrations in the digests of the same sample, was 0.47% for the serum, 5.30% for the placenta, and 8.10% for the colostrum. The mineral concentrations of the tissues (μg/g) were expressed on a dry mass basis, whereas those of the milk and serum were reported as mg/L and µg/L, respectively [6].

### 2.9. Real-Time Quantitative PCR (RT-qPCR)

According to the gene bank (GenBank, http://www.ncbi.nlm.nih.gov/pubmed/), entering the Sus scrofa reference gene glyceraldehyde-3-phosphate dehydrogenase (GAPDH, the reference gene), transporters of Ca, Fe, Zn, Cu genes sequence, primer 5.0 software was used to design the primer according to the principle of the primer design. The primer, designed by Sangon Biotech Co., Ltd, Shanghai, China, and the sequence of primers are shown in Table 2.

Part of the placenta was homogenized in liquid nitrogen to extract the mRNA, as previously described by Xie [14]. Real-time PCR for cDNA templates (experimental extraction of total RNA reverse transcriptase) was carried out using a Luminaris Color HiGreen High ROX (Thermo Scientific) on a Bio-Rad iCycler, according to the manufacturer’s instructions. Each reaction mixture was run in duplicates using optical 384-well reaction plates (BioRad). Each amplification reaction was carried out with 10 μL. Each amplification reaction was carried out with 10 μL of iTaq SYBR Green Supermix (BioRad), mixed with 0.5 μL of each primer, 2 μL of cDNA, and 2 μL of nuclease-free water. The RT-PCR reaction system is shown in Table 3. Negative controls were created by cDNA with nuclease-free water. Initial denaturation for 5 min, followed by 40 cycles of denaturation at 95 °C for 15 s, and primer annealing for 30 s and an extension step at 72 °C for 30 s, were included in the process of amplification. After the amplification, a melt curve analysis with a temperature gradient of 0.1°C/s from 70 °C to 95 °C was performed to confirm that only specific products were amplified. The cDNA templates were pooled and serially diluted six-fold to evaluate the efficiency (E) of the amplification of each primer. Amplification efficiency was calculated from the slope of the standard curve generated by plotting the threshold cycle v. logarithmic values of different DNA concentrations using the equation, E = 10(−1/slope). The Sus scrofa GAPDH gene was used as a reference gene to normalize target gene transcript levels for related trace mineral transporters. Fold changes in mRNA expression levels were calculated using the 2^−ΔΔCt^ method.

### 2.10. Statistical Analysis

The experimental unit for the analysis was the individual sow. All data was expressed as mean ± the standard error of the mean (SEM). Before analysis, all data were examined for normality using histograms and normal distribution plots (UNIVARIATE procedure). Significant differences among treatment meant we were determined by a one-way analysis of variance, followed by Duncan’s multiple-range test (SPSS, 17.0, SPSS Inc., Chicago, IL, USA). For the analysis of reproductive performance, the dietary treatment was considered as the fixed effect the litter was considered as a randomized factor. For the analysis of mineral elements and gene expression, the dietary treatment was considered as the fixed effect the animal was considered as the randomized factor. Probability values <0.05 and <0.01 were considered statistically significant and highly significant, respectively. In addition, significant differences between 9 sows that we selected to collect samples and 25 sows in each group were determined using the Homogeneity of variance test (SPSS, 17.0). The variance of 9 sows that we selected to collect samples and 25 sows is equal when *p* > 0.05.

## 3. Results 

### 3.1. The Feed Intake and Back-Fat Thickness of Sows

The average daily feed intake and back-fat thickness per sow on day 85 of gestation, days 1 and 14 of lactation are summarized in Table 4, in addition, the performance of 9 sows that we selected to collect samples and 25 sows in C, E-L and L-E groups are summarized in Tables S1–S3. As Tables S1–S3 shown, the variance of performance between 9 sows that we selected to collect samples and 25 sows is equal (*p* > 0.05). The average daily feed intake and BF thickness per sow during the experiment period were not influenced by the Ca feeding time (*p* > 0.05).

### 3.2. The Reproductive Performance of Sows

**Figure 1:**
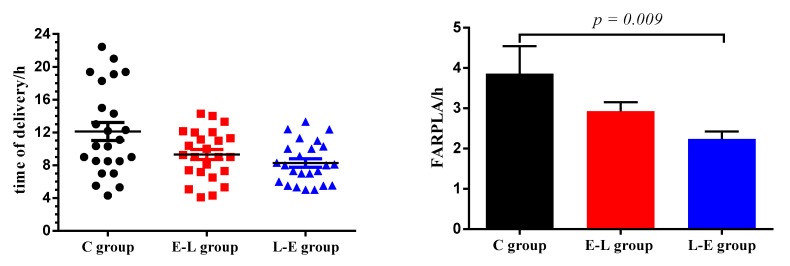
Duration of farrowing and placenta expulsion (FARPLA) of sows, FARPLA is defined as the time interval between the birth of the first piglet and the expulsion of the last placenta. *n* = 25.

The reproductive performances of the sows are shown in Table 5 and Figure 1, in addition, the reproductive performance of 9 sows that we selected to collect samples and 25 sows in C, E-L and L-E groups are summarized in Tables S4–S6. As Tables S4–S6 shown, the variance of reproductive performances between 9 sows that we selected to collect samples and 25 sows in each group is equal (*p* > 0.05). The results showed that, compared with the C group, the FARPLA and stillbirth number/litter decreased significantly in the L-E group (*p* < 0.05). In addition, the times of delivery in the L-E and E-L groups were concentrated between 04:00 and 12:00 every day.

### 3.3. Growth Performance of Suckling Piglets

The growth performance of suckling piglets is shown in Table 6. The results showed that, on average, the weight of piglets from the C group on days 7 and 14 was lower than that of those from the L-E and E-L groups (*p* < 0.05). Moreover, both the E-L and L-E feeding sequences significantly increased the average daily gain of piglets/litter during days 1−14 of lactation (*p* < 0.05). In addition, the survival rate of suckling piglets in the E-L group was significantly higher than that of those in the C group.

### 3.4. The Concentrations of Ca, Cu, Fe, and Zn in the Serum of Sows and Umbilical Cord at Parturition

The concentrations of Ca, Cu, Fe, and Zn in sow serum and umbilical serum are shown in Table 7. Compared with the C group, the controlled Ca feeding times in the L-E and E-L groups increased Ca concentrations in the sow serum and umbilical serum (*p* < 0.05), as well as increasing the concentrations of sow serum Cu and umbilical serum Zn and Fe in the E-L group, and umbilical serum Fe in the L-E group. In contrast, the concentrations of sow serum Zn and umbilical serum Cu in the E-L group and umbilical serum Cu in the L-E group decreased (*p* < 0.05). An upward trend was observed in sow serum Cu in the L-E group (0.05 < *p* < 0.10). 

### 3.5. The Concentrations of Ca, Cu, Fe, And Zn in the Placenta of Sows

The concentrations of Ca, Cu, Fe, and Zn in the placenta are shown in Table 8. Compared with the C group, the L-E and E-L feeding sequences decreased the concentrations of Ca and Fe in the placenta (*p* < 0.05). Additionally, the L-E feeding sequence decreased placental Zn and Cu (*p* < 0.05). 

### 3.6. mRNA Expression of Mineral Element Transporters in the Placenta of Sows

**Figure 2:**
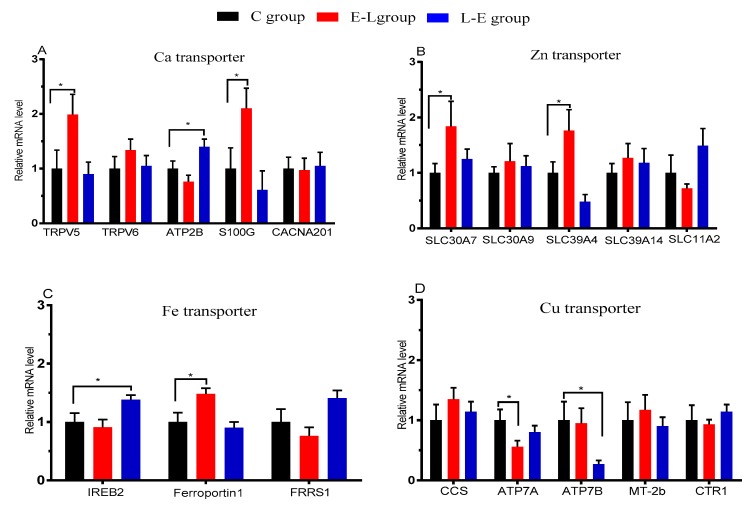
mRNA expression of Ca, Cu, Fe, and Zn transporters in the placenta of sows; *n* = 9. Statistical significances were set at * *p* < 0.05.

The mRNAs of Ca, Zn, Cu, and Fe transporters in the placenta are shown in Figure 2. Experiments on the genes involved in mineral element transport showed that the E-L feeding sequence up-regulated the mRNA expression levels of TRPV5, S100G, SLC30A7, SLC39A4, and Ferroportin1 (*p* < 0.05) in the placenta when compared with the C group, while it down-regulated the level of ATP7A (*p* < 0.05). The L-E feeding sequence up-regulated the placental mRNA expression levels of ATP2B and IREB2 (*p* < 0.05), while it down-regulated the levels of ATP7B (*p* < 0.05), compared with the C group.

### 3.7. The Concentration of Ca, Cu, Fe, and Zn in the Colostrum of Sows

The concentrations of Ca, Cu, Fe, and Zn in the colostrum are shown in Table 9. The concentrations of Ca, Zn, and Cu in the colostrum were all affected by varying the Ca feeding time. The concentrations of Zn and Cu increased, while that of Ca decreased, in the E-L and L-E groups, compared with the C group (*p* < 0.05). 

### 3.8. The Concentration of Ca, Zn, Cu, and Fe in the Serum and Milk of Sows on Day 7 of Lactation

**Figure 3:**
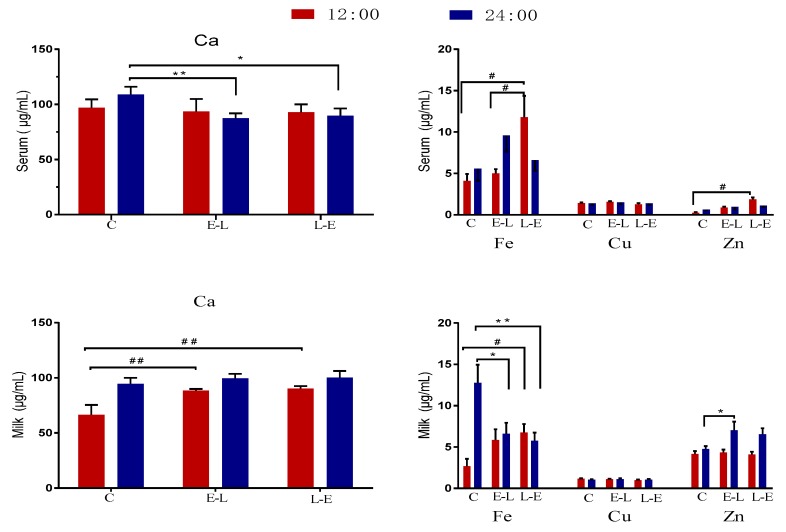
The concentration of Ca, Zn, Cu, and Fe in the serum and milk of sows on day 7 of lactation. Data are presented as mean ± SEM, *n* = 9. Statistical significances were set at ^#^ *p* < 0.05 and ^##^ *p* < 0.01 at 12:00 and at * *p* < 0.05 and ** *p* < 0.01 at 24:00.

The concentrations of Ca, Zn, Cu, and Fe in the serum and milk of sows on day 7 of lactation are shown in Figure 3. Compared with the C group, at 12:00, the E-L group displayed increased milk Ca (*p* = 0.004) and demonstrated an upward trend in milk Fe (*p* < 0.05). Additionally, at 24:00, the E-L group displayed increased milk Zn and an upward trend in serum Fe, while serum Ca and milk Fe decreased (*p* < 0.05). Similarly, the L-E feeding sequence increased serum Fe, serum Zn, milk Ca, and milk Fe at 12:00 (*p* < 0.05), while the group displayed an upward trend in milk Zn, and decreased serum Ca and milk Fe, at 24:00 (0.05 < *p* < 0.10).

## 4. Discussion

Metabolic chronophysiology is of high importance with regards to eating time effects on the health of livestock [5], while the effect of Ca feeding time during late pregnancy and lactation on the performance of sows has never been studied. The study showed that under isocaloric conditions per kilogram of live metabolic weight, feeding time did not influence sow body weight (BW) and BW gain either during gestation or lactation [9], because the nutritional needs of the sows for energy was not compromised at the different feeding times. Similar results also appeared in this experiment; the fact that BF gain and loss did not differ with reference to feeding time in this study may be due to the similar feed intake observed.

The Ca requirements of sows increase during late gestation and lactation [4]. On the one hand, lower Ca utilization or insufficient Ca in the diet will lead to Ca deficiency, and inadequate mineral supplies may prolong delivery time, increase the number of stillbirths, and decrease the vigor of piglets [4]. It has been demonstrated that feeding with different amounts of Ca during late pregnancy does not affect newborn birth weight or litter size [21]. In the present study, feeding Ca to sows at 15:00 during late pregnancy significantly decreased the FARPLA of sows. Furthermore, the time of sow delivery in the L-E group was concentrated between 04:00−12:00 daily. Calcium is involved in muscle contraction during the delivery of sows. In addition, evening feeding improved nutrient digestibility [5]. Therefore, we speculated that feeding Ca at 15:00 may improve the digestibility of Ca, which is beneficial for muscle contraction during sow delivery, but the specific mechanism needs further study. In addition, the study showed that there was a significant increase in the rate of stillbirths as the delivery time increased [22]. This result is consistent with our experimental results, thus, it was believed that the shorter FARPLA of sows in the L-E group was the main reason for the lower rate of stillbirths. On the other hand, the average daily weight gain (ADG) of suckling piglets from sows fed low Ca diets (0.38% of Ca) or fed high Ca diets (1.12% of Ca) was greater than that of those from sows fed standard concentrations of Ca [2]. Additionally, the study showed that the time of feeding was a determining factor in the weight gain of the mammal [7], while feeding sows a daily meal at 15:30 had no difference in the ADG of suckling piglets compared with the control [9]. Therefore, it was speculated that Ca feeding time may affect the Ca content in the blood and milk of sows, and that the utilization of Ca in suckling piglets was improved, which may be the main reason for increasing the ADG of piglets.

The placenta is an extremely important organ that is responsible for the exchange of nutrients, gases, wastes, and biologically active substances between the maternal and fetal system [23]. Umbilical serum mineral element concentrations have been used to monitor the fetal mineral element status. Additionally, the transfer of minerals through the placental barrier occurs via several different transporters [24]. Therefore, it can be inferred that the concentration of minerals in the sow serum, placenta, and umbilical serum are affected directly by a placental mineral transporter expression. In the present study, placental Ca, Fe, Zn, and Cu transporters were affected by Ca feeding time during late pregnancy.

Ca plays an important role in embryo implantation and development, and the functioning of the placenta [25]. Ca passes through the placental barrier via several different Ca channels and transporters. To date, studies have shown that TRPV6, TRPV5, S100G, and ATP2B1 are expressed in placental trophoblasts in pigs [26]. One paper reports that serum Ca is correlated with dietary Ca at farrowing [27], and sows fed the low Ca diet in late gestation will reduce the serum Ca of sows, and the highest serum Ca level is seen in the neonatal piglets from sows fed a high Ca diet at birth [21]. In addition, feeding Ca at 06:00 and 15:00 during late pregnancy affects mRNA expression of the placental circadian clock in pigs [13]. Consistent with these observations, the present study shows that the umbilical serum Ca increased significantly in both the E-L and L-E groups. Similarly, sow serum Ca concentrations increased significantly. In contrast, placental Ca concentrations decreased significantly. Consistent with these observations, the mRNA expression levels of Ca transporters in the placenta, including TRPV5, S100G, and ATP2B, were up-regulated significantly in the E-L and L-E groups. Therefore, it is speculated that Ca feeding time to sows increased the utilization of calcium for sows, and Ca feeding time may affect the mRNA expression of placental transporters of Ca by regulating the placental mRNA expression of Period1 (PER1), Period2 (PER2), and Clock gene (CLOCK). Higher umbilical serum Ca might be beneficial to fetal growth and development [28], and lower placental Ca concentrations may reduce the possibility of placental calcification. Additionally, higher sow serum Ca during delivery may contribute to reducing the risk of hypocalcemic responses. These results may account for the reduction of stillbirths in this study.

Zn is an essential trace mineral and plays a significant role in the metabolism, growth, and development of the body. Two protein families are involved in placental Zn transport, the ZnT proteins (Slc30), and the ZIP proteins (Slc39) [29]. The present study showed that feeding Ca to sows at 06:00 significantly increased umbilical serum Zn. In contrast, the concentration of Zn in the sow serum and placenta in the E-L group decreased. Consistent with these observations, the mRNA expression of the Zn transporters in the placenta, including Slc39A4 and Slc30A7, were significantly upregulated in the E-L group. Therefore, it was believed that the time of feeding Ca to sows regulated placental Zn transport by regulating the mRNA expression level of transporters. Zn is important to fetal neurological development and is related to the developmental status during infancy. Therefore, increased umbilical serum Zn may contribute to fetal development. 

As an essential micronutrient, Fe plays an important role in the development of the placenta and fetus during the fetal and neonatal period. A number of transporters, including transferrin receptor1 (TFR1), DMT1, ZIP14, ferroportin 1 (FPN1), Fe^3+^ che/ate reductase 1 (FRRS1), and Fe responsive element binding protein (IRE-BP), are involved in placental Fe transport [30,31]. The present study shows that feeding Ca to sows at 06:00 or 15:00 significantly increased the umbilical serum Fe concentration and decreased placental Fe. Consistent with these observations, the mRNA expression of REB2 and FPN1 increased significantly in the L-E and E-L groups, respectively. It was believed that the time of feeding Ca to sows affected umbilical serum Fe by regulating placental mRNA expression of REB2 and FPN1. It is reported that anemia and Fe deficiency during pregnancy are associated with high placental weights and high ratios of placental weight to birth weight [32]. Therefore, a higher concentration of Fe in the umbilical cord blood may contribute to fetal development.

A series of enzymes and carrier proteins are involved in placental Cu transport. The main role of ATP7A/ATP7B is the catalyzation of adenosine triphosphate (ATP) decomposition to supply energy and promote the excretion of Cu [33]. This study shows that umbilical serum Cu decreased significantly in both the E-L and L-E groups. Consistent with these observations, placental mRNA expression of Cu transporters, including ATP7A and ATP7B, were significantly down-regulated in the E-L and L-E groups. These results demonstrated that the time of feeding Ca to sows during late pregnancy significantly decreased umbilical serum Cu by down-regulating the placental mRNA expression of ATP7A/ATP7B.

Colostrum synthesis is largely initiated before parturition, and mineral composition of colostrum can be influenced by the mineral needs of the pregnant sow and by dietary mineral supplementation [3]. In addition, the interrelationships between mineral elements are complex. Research has indicated that increasing Ca intake can reduce the bioavailability of Zn, Cu, and Fe in animals, but that these effects may be dose-dependent [21]. In the present study, an interesting observation was that colostral Ca decreased significantly in the E-L and L-E groups, although sow serum Ca increased. In contrast, colostral Cu and Zn increased significantly in both the E-L and L-E groups. It is believed that feeding Ca to sows at 06:00 or 15:00 during late pregnancy mainly promotes the utilization of Ca by fetus; however, the specific mechanism needs further research.

There were some changes in the Ca, Fe, and Zn contents of serum and milk observed between the day and night on day 7 of lactation, in the present study. On the one hand, in the present study, milk Ca increased significantly at 12:00 in both the E-L and L-E groups. In contrast, serum Ca decreased significantly at 24:00 in the E-L and L-E groups. In addition, one study shows that there was an upwards trend in the milk Ca of sows fed a high Ca diet, compared to that from other groups [2]. Therefore, it is believed that maternal Ca feeding at 06:00 or 15:00 may promote the utilization of Ca during the night. On the other hand, in the present study, maternal Ca feeding at 15:00 significantly increased milk Fe during the day and decreased milk Fe during the night. In addition, maternal feeing Ca at 15:00 significantly increased milk Zn during the day and night. While it has been reported that a high Ca diet for two weeks does not inhibit the Fe absorption or alter the Fe status of young piglets [34], another study found that mRNA expression of genes involved in Fe absorption and metabolism in pigs can be regulated by the time of feeding Fe [35]. In addition, placental mRNA expression of PER1, PER2, and CLOCK can be affected by Ca feeding time [13]. Therefore, it was speculated that the gene expression of Ca, Fe, and Zn transporters in mammary gland tissue may be related to PER1, PER2, and CLOCK, and the mutual antagonism between Ca and Zn for their absorption at the mammary epithelial cell may be weakened by changing the time of feeding. For technical reasons, the mRNA expressions of mineral element transporters and of PER1, PER2, and CLOCK in the mammary glands were not measured in the present study and these specific mechanisms need to be further studied.

## 5. Conclusions

In conclusion, compared to conventional feeding regimes, maternal Ca feeding at 15:00 during late pregnancy and lactation may decrease stillbirth and FARPLA and improve the growth performance of suckling piglets by altering the mineral element (including Ca, Fe, Zn, and Cu) metabolism in the umbilical serum and milk.

## Figures and Tables

**Table 1:** Composition and nutrient levels of diets (air-dry basis) (%).

Items	Late Pregnancy Diet	Lactation Diet
Ingredients (%)		
Ground corn	46.37	55.05
Soybean meal	10.43	9.00
Wheat bran	25.00	20.00
Wheat	10.92	8.00
Zeolite powder	3.55	4.26
CaHPO_4_	1.27	1.27
CaCO_3_	0.00	0.00
Lysine (54.6%)	0.44	0.40
Phytase ^1^	0.01	0.01
Premix ^2^	2.02	2.02
Total	100.00	100.00
Chemical composition % ^3^		
DE (kcal/kg)	3000.72	3418.67
Ca (%)	0.30	0.30
Total phosphorus (P) (%)	0.71	0.71
Available P (%)	0.50	0.50
Crude fat (%)	6.70	4.82
Crude fiber (%)	4.28	2.77
Ash (%)	4.59	3.33

^1^ Phytase, 1000 FTU/kg (FTU: Fytase Unit), from Axtra^®^ PHY (a new generation fungal phytase, 6-phytase from *Buttiauxella* sp., expressed in *Trichoderma reesei*), Danisco Animal Nutrition, DuPont Industrial Biosciences. ^2^ The vitamin–mineral premix provided the following per kilogram of diet: 30 mg of antioxidant, 6000 IU (International Unit) of vitamin A, 3000 IU of vitamin D3, 20 IU of vitamin E, 1.8 mg of vitamin K3, 2.0 mg of thiamine, 6.0 mg of riboflavin, 4.0 mg of pyridoxine, 0.02 mg of vitamin B12, 26.0 mg of niacin, 18.0 mg of pantothenic acid, 3.2 mg of folic acid, 0.4 mg of biotin, 27 mg of Mg as MgSO_4_·H_2_O. ^3^ The nutrient levels were calculated values.

**Table 2:** Sequence of primers for real time-polymerase chain reaction.

Target Gene	Gene Bank ID	Nucleotide Sequence of Primers (5′–3′)	Size (bp)
Ca transporter			
ATP2B1	XM_021091182.1	F:CTGGTTGGATTGAAGGTGCT	123
		R:GCTCCTGCTCAATTCGACTC	
TRPV6	XM_021078898.1	F:CTAACAAGCTGGGCCATTTC	119
		R:GCTGTACATGAAGGGCAGGT	
TRPV5	XM_021078897.1	F:CATGTACTTTGCCCGAGGAT	125
		R:GGCAAATCCCAGAGTAACCA	
CACNA2D1	XM_021102233.1	F:TGTACCTGGATGCACTGGAA	122
		R:TCCCATCACACCAAGAATCA	
S100G	NM_214140.2	F:TCCTGCAGAACTGAAGAGCA	133
		R:TAGGGTTCTCGGACCTTTCA	
Zn transporter			
Slc30A7	XM_005655460.2	F:CCTCTTTAACGGTGCTCTCG	119
		R:CATGAAAGTGTCCGTGTCCA	
Slc30A9	NM_001137632.1	F:ATTAGGCGTGGTCTCAGCAT	119
		R:TTACTGACGGGTCGTTCTCC	
Slc39A4	XM_021090449.1	F:AGCTCAGCCAGTCAGAGAGG	123
		R:TGACGTAGTGGGTAGCAGCA	
Slc39A14	XM_005657235.3	F:AGGATGAAAGGAAGGGCAGT	114
		R:TACCCGATCTGGATCTGTCC	
Fe transporter			
Ferroportin1	XM_013984335.2	F:TGGGTGAGAAAGACCCTGAC	109
		R:TAGGAGACCCATCCATCTCG	
IREB2	NM_001167781.1	F:CTCTTCCCGGACAGTGTTGT	124
		R:GAGAAACTGGCAGACCAAGC	
FRRS1	XM_021088483.1	F:CCTGCGTCTTCTTGTCCTTC	120
		R:CATCATCATCACCCATCCAA	
Cu transporter			
CCS	NM_001001866.1	F:CTTCAGGATGGAGGATGAGC	119
		R:TCCCGGTGATCTTGGATAAG	
ATP7A	XM_013990938.2	F:TCTGGCAGCACTGTTATTGC	116
		R:GCCTCCTCCACAAGTTTGAC	
ATP7B	XM_021065286.1	F:TATGACCCTTCCTGCGTCTC	121
		R:ACCTGGCATCTGTTCCTGTC	
MT-2b	XM_003355808.4	F:TCCTGCAAATGCAAAGACTG	119
		R:CACTTGTCCGAGGCTCCTT	
CTR1	NM_214100.3	F:CGCAAATCACAAGTCAGCAT	129
		R:CACTGTCTGCAGGAGGTGAG	
Others			
Slc11A2(DMT1)	XM_021081710.1	F:GTCTCAGTCTTTGCCGAAGC	120
		R:CACAGCCAGTGTCGAGTTGT	
GAPDH	XM_021091114.1	F:GTCGGTTGTGGATCTGACCT	120
		R:GTCCTCAGTGTAGCCCAGGA	

**Table 3:** Real time-polymerase chain reaction system.

Item	μL
SYBR Green Supermix ^1^	5
Forward Primer	0.5
Reverse primer	0.5
cDNA template	2
Nuclease-free water.	2
Total	10

^1^ SYBR Green: SYBR Green Nucleic Acid Gel Stains.

**Table 4:** Effects of Ca feeding time on sow performance (*n* = 25).

Item	Dietary Treatment			*p*-Value
	C Group	E-L Group	L-E Group	
Parity	4.05 ± 0.350	4.00 ± 0.297	4.38 ± 0.344	0.677
Daily feed intake of per sow, kg				
85th–114th day of pregnancy	2.09 ± 0.148	2.26 ± 0.054	2.24 ± 0.075	0.435
1st to 14th day of lactation	4.64 ± 0.349	4.89 ± 0.432	4.99 ± 0.459	0.826
Sow backfat thickness, mm				
On 85th day of pregnancy	15.89 ± 0.491	15.58 ± 0.491	15.47 ± 0.509	0.891
On 1st day of lactation	16.58 ± 0.745	15.84 ± 0.584	16.74 ± 0.648	0.713
On 14th day of lactation	14.58 ± 0.652	14.26 ± 0.728	14.74 ± 0.732	0.915
BF gain or loss, mm				
85th day of pregnancy to farrowing	0.69 ± 0.737	0.95 ± 0.594	1.79 ± 0.379	0.537
1st–14th day of lactation	−2.00 ± 0.562	−1.58 ± 0.636	−2.00 ± 0.341	0.857

Data are presented as mean ± standard error of the mean (SEM), *n* = 25. The control (C) group = fed extra Ca at both 06:00 and 15:00, the earlier-later (E-L) group = fed extra Ca at 06:00, and the laer-earlier (L-E) group = fed extra Ca at 15:00, the same as below.

**Table 5:** The reproductive performance of sows (*n* = 25).

Item	Dietary Treatment			*p*-Value
	C Group	E-L Group	L-E Group	
Litter size, *n*	12.14 ± 0.517	11.85 ± 0.460	12.5 ± 0.489	0.651
Number born alive, *n*	10.38 ± 0.374 ^a^	10.45 ± 0.444 ^ab^	11.4 ± 0.358 ^b^	0.134
Number of stillbirths, *n*	1.71 ± 0.293	1.1 ± 0.260	0.95 ± 0.198	0.087
IUGR, *n* ^1^	0.23 ± 0.136	0.05 ± 0.040	0.15 ± 0.130	0.548
Birth weight, kg	1.36 ± 0.045	1.48 ± 0.052	1.40 ± 0.033	0.178

^1^ Neonatal piglets with birth weights close to the mean birth weight (±0.5 standard deviation (SD)) were identified as having normal birth weights, whereas piglets with at least 1.5 SD lower birth weight were defined as suffering from intra-uterine growth restriction (IUGR). ^a,b^ Within a row, means with different superscripts differ (*p* < 0.05) by *t*-test.

**Table 6:** Growth performance of suckling piglets (*n* = 20).

Item	Dietary Treatment			*p*-Value
	C Group	E-L Group	L-E Group	
Number of piglets (*n*) on:				
1st day of life	11.6 ± 0.210	11.20 ± 0.263	11.63 ± 0.283	0.436
14th day of life	9.11 ± 0.285	9.75 ± 0.216	9.30 ± 0.230	0.17
Survival rate, %	80.37 ± 2.728 ^b^	88.67 ± 2.152 ^a^	83.23 ± 2.305 ^ab^	0.049
Body weight (kg) on:				
1st day of life	1.34 ± 0.038	1.42 ± 0.049	1.40 ± 0.034	0.403
7th day of life	2.39 ± 0.111 ^b^	2.68 ± 0.074 ^a^	2.87 ± 0.091 ^a^	0.002
14th day of life	3.57 ± 0.132 ^b^	4.35 ± 0.098 ^a^	4.62 ± 0.122 ^a^	<0.001
Daily gains, kg				
1–7	0.15 ± 0.015 ^b^	0.18 ± 0.012 ^ab^	0.21 ± 0.015 ^a^	0.028
7–14	0.17 ± 0.010 ^b^	0.24 ± 0.006 ^a^	0.25 ± 0.011 ^a^	<0.001
1–14	0.16 ± 0.009 ^b^	0.2 ± 0.008 ^a^	0.23 ± 0.010 ^a^	<0.001

^a,b^ Within a row, means with different superscripts differ (*p* < 0.05) by *t*-test.

**Table 7:** The concentrations of Ca, Cu, Fe, and Zn in the serum of sows and umbilical cord at parturition (*n* = 9).

Item	Dietary Treatment			*p*-Value
	C Group	E-L Group	L-E Group	
Sow serum on farrowing day				
Ca (mmol/L)	2.22 ± 0.06 ^b^	2.43 ± 0.07 ^a^	2.42 ± 0.05 ^a^	0.045
Cu (µg/mL)	1.24 ± 0.06 ^b^	1.47 ± 0.09 ^a^	1.42 ± 0.06 ^ab^	0.087
Fe (µg/mL)	1.23 ± 0.387	2.75 ± 0.631	2.07 ± 0.552	0.165
Zn (µg/mL)	1.70 ± 0.44 ^a^	0.51 ± 0.14 ^b^	1.03 ± 0.35 ^ab^	0.090
Umbilical cord serum				
Ca (mmol/L)	2.96 ± 0.23 ^a^	4.55 ± 0.14 ^c^	3.67 ± 0.22 ^b^	0.001
Cu (µg/mL)	0.50 ± 0.23 ^a^	0.08 ± 0.01 ^b^	0.05 ± 0.02 ^b^	0.021
Fe (µg/mL)	1.70 ± 0.41 ^b^	5.72 ± 0.63 ^a^	5.40 ± 0.98 ^a^	0.001
Zn (µg/mL)	1.33 ± 0.23 ^b^	3.75 ± 1.16 ^a^	2.42 ± 0.68 ^ab^	0.110

^a,b,c^ Within a row, means with different superscripts differ (*p* < 0.05) by *t*-test.

**Table 8:** The concentrations of Ca, Cu, Fe, and Zn in the sows’ placenta (*n* = 9).

Item	Dietary Treatment			*p*-Value
	C Group	E-L Group	L-E Group	
Ca (mg/100 g)	36.58 ± 3.28 ^a^	29.41 ± 1.60 ^b^	27.65 ± 1.96 ^b^	0.036
Cu (mg/100 g)	0.049 ± 0.01 ^a^	0.052 ± 0.01 ^a^	0.028 ± 0.003 ^b^	0.041
Fe (mg/100 g)	4.17 ± 0.93 ^a^	2.21 ± 0.44 ^b^	2.17 ± 0.29 ^b^	0.048
Zn (mg/100 g)	0.50 ± 0.05 ^a^	0.40 ± 0.04 ^ab^	0.34 ± 0.04 ^b^	0.046

^a,b^ Within a row, means with different superscripts differ (*p* < 0.05) by *t*-test.

**Table 9:** The concentration of Ca, Cu, Fe, and Zn in the colostrum of sows (*n* = 9).

Item	Dietary Treatment			*p*-Value
	C Group	E-L Group	L-E Group	
Ca (g/L)	59.93 ± 9.407 ^a^	34.06 ± 2.590 ^b^	33.36 ± 2.322 ^b^	0.017
Cu (µg/mL)	1.62 ± 0.234 ^b^	3.27 ± 0.821 ^a^	3.17 ± 0.406 ^a^	0.016
Fe (µg/mL)	9.00 ± 2.810	8.38 ± 3.300	6.62 ± 1.690	0.811
Zn (µg/mL)	10.07 ± 1.603 ^b^	16.67 ± 1.407 ^a^	14.08 ± 0.774 ^a^	0.010

^a,b^ Within a row, means with different superscripts differ (*p* < 0.05) by *t*-test.

## Supplementary Materials

The following are available online at https://www.mdpi.com/2076-2615/9/6/337/s1, Table S1: The performance of sows in the C group, Table S2: The performance of sows in E-L group, Table S3: The performance of sows in L-E group, Table S4: The reproductive performance of sows in C group, Table S5: The reproductive performance of sows in E-L group, Table S6: The reproductive performance of sows in L-E group.

## References

[1] Mahan D.C., Watts M.R., St-Pierre N. (2009). Macro- and micromineral composition of fetal pigs and their accretion rates during fetal development. J. Anim. Sci..

[2] Miller M.B., Hartsock T.G., Erez B., Douglass L., Alston-Mills B. (1994). Effect of dietary calcium concentrations during gestation and lactation in the sow on milk composition and litter growth. J. Anim. Sci..

[3] Peters J.C., Mahan D.C., Wiseman T.G., Fastinger N.D. (2010). Effect of dietary organic and inorganic micromineral source and level on sow body, liver, colostrum, mature milk, and progeny mineral compositions over six parities. J. Anim. Sci..

[4] Mahan D.C. (1990). Mineral-nutrition of the sow—A review. J. Anim. Sci..

[5] Nikkhah A. (2012). Eating Time Modulations of Physiology and Health: Life Lessons from Human and Ruminant Models. Iran. J. Basic Med. Sci..

[6] Deng Y., Wang Z.V., Gordillo R., An Y., Zhang C., Liang Q., Yoshino J., Cautivo K.M., De Brabander J., Elmquist J.K. (2017). An adipo-biliary-uridine axis that regulates energy homeostasis. Science.

[7] Arble D., Bass J., Laposky A., Vitaterna M.H., Turek F.W. (2009). Circadian timing of food intake contributes to weight gain. Obesity.

[8] Wang J., Patterson R., Ang A., Emond J.A., Shetty N., Arab L. (2014). Timing of energy intake during the day is associated with the risk of obesity in adults. J. Hum. Nutr. Diet..

[9] Manu H., Lee S.H., Ren P., Pangeni D., Yang X., Baidoo S.K. (2019). Effects of time of feeding during gestation on sow’s performance1. J. Anim. Sci..

[10] Wu X., Xie C., Guo X., Long C., Zhang T., Gao T., Yin Y. (2017). A maternal two-meal feeding sequence with varying crude protein affects milk lipid profile in a sow-piglet model. Sci. Rep..

[11] Markowitz M.E., Arnaud S., Rosen J.F., Thorpy M., Laximinarayan S. (1988). Temporal interrelationships between the circadian rhythms of serum parathyroid hormone and calcium concentrations. J. Clin. Endocrinol. Metab..

[12] Lin X., Liu Y., Xie C., Wu X., Yin Y. (2018). Circadian rhythms and dynamic dietary calcium feeding affect laying performance, calcium and phosphorus levels in laying hens. Biol. Rhythm Res..

[13] Gao L.M., Xie C.Y., Zhang T.Y., Wu X., Yin Y.L. (2018). Maternal supplementation with calcium varying with feeding time daily during late pregnancy affects lipid metabolism and transport of placenta in pigs. Biochem. Biophys. Res. Commun..

[14] Xie C., Wu X., Long C., Wang Q., Fan Z., Li S., Yin Y. (2016). Chitosan oligosaccharide affects antioxidant defense capacity and placental amino acids transport of sows. BMC Vet. Res..

[15] Fan Z., Xiao Y., Chen Y., Wu X., Zhang G., Wang Q., Xie C. (2015). Effects of catechins on litter size, reproductive performance and antioxidative status in gestating sows. Anim. Nutr..

[16] Wu X., Yin Y.L., Liu Y.Q., Liu X.D., Liu Z.Q., Li T.J., Huang R.L., Ruan Z., Deng Z.Y. (2012). Effect of dietary arginine and n-carbamoylglutamate supplementation on reproduction and gene expression of enos, vegfa and plgf1 in placenta in late pregnancy of sows. Anim. Reprod. Sci..

[17] Van Rens B.T., van der Lende T. (2004). Parturition in gilts: Duration of farrowing, birth intervals and placenta expulsion in relation to maternal, piglet and placental traits. Theriogenology.

[18] Wu G., Bazer F.W., Wallace J.M., Spencer T.E. (2006). Board-invited review: Intrauterine growth retardation: Implications for the animal sciences. J. Anim. Sci..

[19] Che L., Hu L., Liu Y., Yan C., Peng X., Xu Q., Wang R., Cheng Y., Chen H., Fang Z. (2016). Dietary nucleotides supplementation improves the intestinal development and immune function of neonates with intra-uterine growth restriction in a pig model. PLoS ONE.

[20] Wan D., Zhang Y.M., Wu X., Lin X., Shu X.G., Zhou X.H., Du H.T., Xing W.G., Liu H.N., Li L. (2018). Maternal dietary supplementation with ferrous n-carbamylglycinate chelate affects sow reproductive performance and iron status of neonatal piglets. Animal.

[21] Tan F. Impact of Dietary Calcium and Phosphorus on Sow Reproductive Performance and Bone Development in Piglets. https://pdfs.semanticscholar.org/06fd/bf31344a7b720edd64e48d09cab496da277d.pdf.

[22] Van Dijk A.J., van Rens B.T., Van D.L.T., Taverne M.A. (2005). Factors affecting duration of the expulsive stage of parturition and piglet birth intervals in sows with uncomplicated, spontaneous farrowings. Theriogenology.

[23] Lager S., Powell T.L. (2012). Regulation of nutrient transport across the placenta. J. Pregnancy.

[24] Mahan D.C., Vallet J.L. (1997). Vitamin and mineral transfer during fetal development and the early postnatal period in pigs. J. Anim. Sci..

[25] Baczyk D., Kingdom J.C., Uhlen P. (2011). Calcium signaling in placenta. Cell Calcium.

[26] Choi Y., Seo H., Kim M., Ka H. (2009). Dynamic expression of calcium-regulatory molecules, trpv6 and s100g, in the uterine endometrium during pregnancy in pigs. Biol. Reprod..

[27] Wuryastuti H., Stowe H.D., Miller E.R. (1991). The influence of gestational dietary calcium on serum 1, 25-dihydroxycholecalciferol in sows and their pigs. J. Anim. Sci..

[28] Fahimeh K., Reza S.M., Ali M., Javad S.R. (2016). Determination of maternal serum zinc, iron, calcium and magnesium during pregnancy in pregnant women and umbilical cord blood and their association with outcome of pregnancy. Mater. Sociomed..

[29] Helston R.M., Phillips S.R., McKay J.A., Jackson K.A., Mathers J.C., Ford D. (2007). Zinc transporters in the mouse placenta show a coordinated regulatory response to changes in dietary zinc intake. Placenta.

[30] Zhao N., Gao J., Enns C.A., Knutson M.D. (2010). Zrt/irt-like protein 14 (zip14) promotes the cellular assimilation of iron from transferrin. J. Biol. Chem..

[31] Gunshin H., Fujiwara Y., Custodio A.O., Direnzo C., Robine S., Andrews N.C. (2005). Slc11a2 is required for intestinal iron absorption and erythropoiesis but dispensable in placenta and liver. J. Clin. Investig..

[32] Steer P. (1991). The effect of maternal anaemia and iron deficiency on the ratio of fetal weight to placental weight. Br. J. Obstet. Gynaecol..

[33] Mercer J.F., Barnes N., Stevenson J., Strausak D., Llanos R.M. (2003). Copper-induced trafficking of the cu-atpases: A key mechanism for copper homeostasis. Biometals.

[34] Wauben I.P., Atkinson S.A. (1999). Calcium does not inhibit iron absorption or alter iron status in infant piglets adapted to a high calcium diet. J. Nutr..

[35] Zhang Y., Wan D., Zhou X., Long C., Wu X., Li L., He L., Huang P., Chen S., Tan B. (2017). Diurnal variations in iron concentrations and expression of genes involved in iron absorption and metabolism in pigs. Biochem. Biophys. Res. Commun..

