# Supplementary material for: The time of Calcium Feeding Affects the Productive Performance of Sows
Source: Animals (Basel). 2019 Jun 10;9(6):337. doi: 10.3390/ani9060337 (PMC6617234; doi:10.3390/ani9060337)
Supplement: Supplementary file 1 [file animals-09-00337-s001.pdf]

Supplementary material

Sows (n = 9) that we selected to collect samples in each group are considered as the A group, and all sows (n = 25) in each group are considered as the B group. We compared the performance and reproductive performance of sows between A and B groups. All data was expressed as mean ± the standard error of the mean (SEM). Significant differences among treatment means were determined using the Homogeneity of variance test (SPSS, 17.0). The variance of each group is equal when $p > 0.05$; the variance of each group is not equal when $p < 0.05$. The results are as follows:

Table S1. The performance of sows in the C group

	groups		*p*-value
	A group	B group	
Parity	4.25 ± 0.526	4.05 ± 0.350	0.761
Daily feed intake of per sow, kg			
85th–114th day of pregnancy	2.043 ± 0.095	2.09 ± 0.148	0.691
1st–14th day of lactation	4.68 ± 0.225	4.64 ± 0.349	0.809
Sow BF, mm			
On 85th day of pregnancy	15.87 ± 0.350	15.89 ± 0.491	0.162
On 1st day of lactation	15.88 ± 0.479	16.58 ± 0.745	0.312
On 14th day of lactation	14.00 ± 1.085	14.58 ± 0.652	0.649
BF loss, mm			
1st–14th day of lactation	–1.88 ± 0.789	–2.00 ± 0.562	0.882

A group: Sows (n = 9) that we selected to collect samples in C group; B group: All sows (n = 25) in C group. The variance of each group is equal when $p > 0.05$; the variance of each group is not equal when $p < 0.05$.

Table S2. The performance of sows in E-L group

	group		*p*-value
	A group	B group	
Parity	4.13 ± 0.441	4.00 ± 0.297	0.564
Daily feed intake of per sow, kg			
85th–114th day of pregnancy	2.25 ± 0.095	2.26 ± 0.054	0.543
1st–14th day of lactation	4.73 ± 0.205	4.89 ± 0.432	0.829
Sow BF, mm			
On 85th day of pregnancy	15.49 ± 0.718	15.58 ± 0.491	0.559
On 1st day of lactation	16.10 ± 0.546	15.84 ± 0.584	0.412
On 14th day of lactation	14.4 ± 0.562	14.26 ± 0.728	0.215
BF loss, mm			
1st–14th day of lactation	–1.78 ± 0.472	–1.58 ± 0.636	0.200

A group: Sows (n = 9) that we selected to collect samples in E-L group; B group: All sows (n = 25) in E-L group. The variance of each group is equal when $p > 0.05$; the variance of each group is not equal when $p < 0.05$.

Table S3. The performance of sows in L-E group

	group		p-value
	A group	B group	
Parity	4.5 ± 0.500	4.38 ± 0.344	0.839
Daily feed intake of per sow, kg			
85th—114th day of pregnancy	2.25 ± 0.080	2.24 ± 0.075	0.597
1st–14th day of lactation	4.73 ± 0.205	4.99 ± 0.459	0.802
Sow BF, mm			
On 85th day of pregnancy	15.13 ± 0.639	15.47 ± 0.509	0.351
On 1st day of lactation	16.50 ± 0.534	16.74 ± 0.648	0.274
On 14th day of lactation	14.50 ± 0.597	14.74 ± 0.732	0.242
BF loss, mm			
1st–14th day of lactation	–1.85 ± 0.242	–2.00 ± 0.562	0.205

A group: Sows (n = 9) that we selected to collect samples in L-E group, B group: All sows (n = 25) in L-E group. The variance of each group is equal when $p > 0.05$; the variance of each group is not equal when $p < 0.05$.

Table S4. The reproductive performance of sows in C group

	group		*p*-value
	A group	B group	
Litter size, n	12.20 ± 0.734	12.14 ± 0.517	0.496
Number born alive, n	11.00 ± 0.548	10.38 ± 0.374	0.401
Number of stillbirths, n	2.20 ± 0.374	1.71 ± 0.293	0.405
IUGR, n[1]	0.30 ± 0.213	0.23 ± 0.136	0.760
Birth weight, kg	1.31 ± 0.070	1.36 ± 0.045	0.955
FARPLA, h	3.80 ± 0.300	3.86 ± 0.523	0.198

A group: Sows (n = 9) that we selected to collect samples in C group, B group: All sows (n = 25) in C group. The variance of each group is equal when $p > 0.05$; the variance of each group is not equal when $p < 0.05$.

Table S5. The reproductive performance of sows in E-L group

	group		*p*-value
	A group	B group	
Litter size, n	11.50 ± 0.846	11.85 ± 0.460	0.850
Number born alive, n	10.33 ± 0.954	10.45 ± 0.444	0.664
Number of stillbirths, n	1.21 ± 0.525	1.10 ± 0.260	0.933
IUGR, n	0.10 ± 0.09	0.05 ± 0.040	0.325
Birth weight, kg	1.50 ± 0.070	1.48 ± 0.052	0.207
FARPLA, h	2.93 ± 0.416	2.96 ± 0.277	0.833

A group: Sows (n = 9) that we selected to collect samples in E-L group, B group: All sows (n = 25) in E-L group. The variance of each group is equal when $p > 0.05$; the variance of each group is not equal when $p < 0.05$.

Table S6. The reproductive performance of sows in L-E group

	group		*P*-value
	A group	B group	
Litter size, n	12.00 ± 0.683	12.50 ± 0.489	0.211
Number born alive, n	10.83 ± 0.542	11.40 ± 0.358[b]	0.677
Number of stillbirths, n	0.38 ± 0.201	0.95 ± 0.198	0.306
IUGR, n	0.20 ± 0.100	0.15 ± 0.130	0.738
Birth weight, kg	1.56 ± 0.043	1.40 ± 0.033	0.327
FARPLA, h	2.61 ± 0.280	2.34 ± 0.216	0.366

A group: Sows (n = 9) that we selected to collect samples in L-E group, B group: All sows (n = 25) in L-E group. The variance of each group is equal when $p > 0.05$; the variance of each group is not equal when $p < 0.05$.
